# *Psicologia: Reflexão e Crítica* and its recent contributions to research on multiple United Nations Sustainable Development Goals

**DOI:** 10.1186/s41155-023-00272-0

**Published:** 2023-10-13

**Authors:** Eduardo Remor

**Affiliations:** Institute of Psychology, Social Work, Health and Human Communication, Universidade Federal Do Rio Grande Do Sul, Porto Alegre, Brazil

The United Nations Sustainable Development Goals^1^ (SDGs) are a set of 17 global objectives aimed at addressing social, economic, and environmental challenges by the year 2030. Research initiatives play a vital role in our efforts to develop solutions to these pressing challenges. In research fields such as economics, environmental studies, public health, and urban planning, the relevance of SDGs is well established. However, SDGs are also very important in the field of psychology and applied psychology. This alignment between psychology and the SDGs has the potential to create transformative change and contribute to a more equitable and sustainable world.

As Editor-in-Chief of this multifaceted psychology journal, I am proud to say that our work in *Psicologia: Reflexão e Crítica* [Psychology: Research and Review] significantly contributes towards the SDGs set by the United Nations. Our journal welcomes high-quality papers describing research and reviews on themes under four main fields of psychology: *Developmental Psychology, Psychological Process and Experimental Psychology, Psychological Assessment, and Health Psychology.*

Our coverage on *Developmental Psychology* aligns with SDG 4: Quality Education and SDG 10: Reduced Inequalities. We publish research and studies focusing on cognitive development in children and adolescents, emphasizing how different factors such as biological, interpersonal, educational, social, and cultural affect human psychological development, and which are relevant to education or well-being, across a large number of contexts. This helps educators globally to understand better ways of teaching that cater to diverse developmental needs. Through studies addressing atypical development and developmental psychopathology, researchers can contribute to reducing inequalities by identifying factors that lead to disparities in psychological development. This understanding can inform policies and interventions aimed at providing equal opportunities and support to individuals with diverse developmental needs. The *Psychological Process and Experimental Psychology* section supports SDG 3: Good Health and Well-being as well as SDG 4: Quality Education. Understanding cognitive processes like learning, memory, and problem-solving is crucial for enhancing educational methods and strategies. Research in experimental psychology can contribute to the development of effective teaching techniques and curriculum design, promoting quality education. Our published works often address those topics. This knowledge is crucial for developing effective therapeutic interventions promoting good health and well-being. *Psychological Assessment* content directly feeds into several goals including SDG 3 (Good Health), SDG 10 (Reduced Inequalities), and even indirectly into others like SGD16 (Peace Justice Strong Institutions). The tools we discuss are used worldwide for diagnosing psychological disorders accurately ensuring appropriate treatment, thereby promoting good health; they also help identify disparities in mental healthcare access among various social groups contributing toward reducing inequalities; moreover, they assist legal systems make informed decisions based on psychological evaluations, thus fostering justice and peace. Finally, our focus on *Health Psychology* resonates strongly with several SDGs, particularly SDG 3 (Good Health and Well-being), SDG 4 (Quality Education), and SDG 10 (Reduced Inequalities). Research here explores how psychological factors influence physical and mental wellness providing insights necessary for holistic healthcare approaches fulfilling goal 3; additionally, it investigates humans’ interaction with their environment and this impact on their mental health. It is also related to the SDG 10 Reduced Inequalities, and by examining protective and risk factors in health, researchers in health psychology can identify disparities in health outcomes and work towards reducing inequalities in access to healthcare and health outcomes among different populations.

In this editorial, I would like to showcase six recent articles^2^ that emphasize the importance of research that relates to three SDGs: SDG 3 (Good Health and Well-Being), SDG 4 (Quality Education), and SDG 5 (Gender Equality).

## Interventions to reduce the stigma of mental health at work: a narrative review

Ramírez-Vielma et al. (2023), Vol. 36, number 14.

Stigma towards mental health problems impacts various aspects of life, including access to services and employment. A review of 25 workplace interventions found that most focused on specific disorders and aimed to increase mental health literacy, improve attitudes, and promote help-seeking behaviors. While interventions were generally effective, more research is needed to determine the optimal duration, cultural considerations, and long-term effects of these programs. The study contributes to the SDG3 by promoting mental well-being and highlighting the importance of inclusive work environments.

## Social networks as tools for the prevention and promotion of health among youth

García del Castillo et al. (2020), Vol. 33, number 13.

The rapid evolution of communication technology impacts youth lifestyles and mental health. Researchers analyze the impact of social networks on youth health, focusing on sexual health, body image, and addictions. Social networks are online communities that allow generating individual profiles and express any type of information to interact with other users, to communicate with real-life friends, or to meet others who share similar interests and hobbies. The study highlights the potential of social networks in health prevention and promotion, with positive outcomes in sexual health prevention. However, more research and intervention are needed to promote adequate youth health (e.g., sexual health, body image, especially eating habits and overweight, as well as smoking and alcohol dependence). This article contributes to the SDG 3 by promoting mental health and lifestyle information and skills as “social vaccines” in youth.

## Relation between perceived emotional intelligence and social factors in the educational context of Brazilian adolescents

Vaquero-Diego et al. (2020), Vol. 33, number 1.

Emotional intelligence (EI) plays a crucial role in education, well-being, and social interactions. A recent study examined EI in adolescents from São Paulo City, Brazil, finding significant differences in self-perceived abilities related to gender, age, and parents’ professional activities. Findings suggest that interventions should be personalized according to the gender and age of adolescents. For example, female students may benefit from developing verbal skills and being more competent in articulating feelings, allowing them more resources and awareness to deal with their emotional world. This research contributes to SDG 4 by emphasizing the role of EI in fostering quality education and promoting students’ well-being.

## Gender difference in the effect of cultural distance on academic performance among cross-border students in China

Hu and Cheung (2021), Vol. 34, number 33.

Researchers reveal gender differences in the effect of cultural distance on academic performance among cross-border students in China. Female students face more difficulty adapting to new cultures, impacting their academic performance. The study highlights the need for targeted support and resources for female cross-border students. The study raises awareness for policymakers and universities to build additional resources to support female students’ cultural adaptation, contributing to SDG 4 (Quality Education) and SDG 5 (Gender Equality).

## The Gender Violence-Implicit Association Test to measure attitudes toward intimate partner violence against women

Ferrer-Perez et al. (2020), Vol. 33, number 27.

Researchers developed the Gender Violence-Implicit Association Test (GV-IAT) to assess social attitudes towards intimate partner violence against women (IPVAW). The GV-IAT, designed for Spanish-speaking populations, measures implicit attitudes, reducing the impact of social desirability and response control. This tool may aid in designing preventive actions and public policies to reduce IPVAW, contributing to the SDG5 by addressing gender-based violence.

## The role of physical activity promoting thinking skills and emotional behavior of preschool children

Wang (2022), Vol. 35, number 1.

Physical education boosts critical thinking and emotional well-being in preschoolers, according to a study in Beijing. The research found a strong correlation between age, physical activity, and improved cognitive abilities, with daily physical education classes enhancing mood and sociability. That association was not dependent on the gender of the child. The findings highlight the importance of incorporating physical activity into early childhood education for overall development. This article aligns with SDGs 3 and 4, emphasizing physical education’s role in enhancing preschoolers’ health and education.

## Conclusion

Journals like *Psicologia: Reflexão e Crítica* play a critical role in disseminating research findings and knowledge in the field of psychology. This dissemination helps researchers and practitioners stay informed about the latest developments, which can be applied to address various societal challenges, including those aligned with the SDGs. The research published in *Psicologia: Reflexão e Crítica* highlights the significance of psychological factors that profoundly influence our interactions across diverse contexts, including the workplace, intimate settings, and educational institutions. These findings can guide policies towards achieving the SDGs related to health, education, gender equality, and decent work. By delving into human behavior, attitudes, and motivations, this research provides invaluable insights into the complex challenges faced by societies worldwide.

*Psicologia: Reflexão e Crítica* can support the SDGs by facilitating the dissemination of research, promoting interdisciplinary collaboration, raising awareness, influencing policy, fostering partnerships, and providing a global perspective. Journals like PRC are vital in advancing knowledge and solutions supporting the sustainable development agenda. Researchers and readers can utilize the information and insights published in the journal to contribute to the achievement of the SDGs through their work and actions.

In conclusion, the journal’s comprehensive approach ensures a significant contribution towards achieving multiple SDGs through rigorous scientific exploration within psychology’s broad spectrum. Integrating psychological knowledge with other disciplines is paramount to ensure that the SDGs are achieved efficiently and inclusively.

## Data Availability

Not applicable.

## Abbreviations

SDGs: Sustainable Development Goals
PRC: Psicologia: Reflexão e Crítica

## Footnotes

1 UN Sustainable Development Goals (SDGs) website, https://sdgs.un.org/goals

2 Please note: Article summaries were AI-generated by Springer Nature using the Large Language Model, which were then fact-checked by editors against the original articles.
